# Characterizing airway epithelial cultures grown on permeable inserts with different pore densities

**DOI:** 10.14814/phy2.70744

**Published:** 2026-03-31

**Authors:** T. J. F. Guo, G. K. Singhera, J. Memar Vaghri, W. Y. Liang, J. M. Leung, D. R. Dorscheid

**Affiliations:** ^1^ Centre for Heart Lung Innovation, St. Paul's Hospital Vancouver British Columbia Canada; ^2^ Department of Medicine University of British Columbia Vancouver British Columbia Canada

**Keywords:** air‐liquid interface, airway epithelium, cell culture, permeable inserts

## Abstract

In vitro airway epithelial models, such as air‐liquid interface (ALI) cultures, are widely used for disease modeling, therapeutic and toxicology testing, and studying epithelial‐environmental interactions. However, it remains unknown whether the pore density of the permeable membrane inserts, on which primary human bronchial epithelial cells (HBEC) are grown, influences their morphological and differentiation characteristics. To investigate this, HBEC from seven donors were cultured on inserts with low (4 × 10^6^ pores/cm^2^) and high (1 × 10^8^ pores/cm^2^) pore densities and differentiated for 28 days. ALI cultures on low pore density inserts exhibited greater epithelial thickness, longer cilia, and higher nuclear density compared to those on high pore density inserts. Additionally, periodic‐acid Schiff staining revealed increased mucin production in low pore density cultures. Immunohistochemistry showed significant differences in cytokeratin 5 and FOXJ1 expression between conditions, while MUC5B and β‐tubulin‐IV expression was unchanged. Similarly, transepithelial electrical resistance measurements indicated similar barrier integrity. Our findings indicate that pore density influences HBEC‐derived ALI culture morphology and differentiation, which may contribute to variability across studies and highlight that selecting the appropriate insert is crucial for experimental consistency.

## 1 INTRODUCTION

The airway epithelium serves as a physical and immune barrier against inhaled insults such as particulate matter and pathogens (Guo et al., 2023; Guo, Singhera, et al., 2025; Tam et al., 2011). It consists of various cell types that contribute to its function, with the majority of them being goblet cells that secrete mucus that traps inhaled particles and pathogens, which are then cleared through the mucociliary elevator with rhythmic beating of ciliated cells (Bustamante‐Marin & Ostrowski, 2017). Basal cells function as progenitor cells, capable of differentiating into other types of epithelial cells to maintain and repair the epithelium (Deprez et al., 2020). Junctional complexes such as tight and adherens junctions help to regulate paracellular solute movement and cell–cell adhesion, respectively (Ganesan et al., 2013). Dysfunction of the airway epithelium is associated with several respiratory diseases, including asthma, cystic fibrosis, and chronic obstructive pulmonary disease (COPD), contributing to their onset and progression (Calvén et al., 2020; Rose et al., 2018).

In vitro models of the airway epithelium are valuable tools for testing and development of therapeutics, studying disease mechanisms, and exploring the interactions between the epithelium and environmental agents (Guo, Liang, et al., 2025; Karp et al., 2002). Air‐liquid interface (ALI) cultures have been developed to replicate the airway epithelium's pseudostratified ciliated phenotype (Bhowmick & Gappa‐Fahlenkamp, 2016). Studies have shown that differentiated ALI cultures closely resemble in vivo epithelia in both structure, as seen in histological analysis, and function, including transepithelial transport, barrier integrity, and innate immune responses (Assou et al., 2023; Lee et al., 2023; Leung et al., 2020; Prescott et al., 2023). Additionally, their transcriptomic profiles align closely with those of in vivo epithelial cells (Pezzulo et al., 2011), underscoring the ability of ALI cultures to accurately recapitulate the structure and function of the in vivo airway.

To establish an ALI culture, human bronchial epithelial cells (HBEC) are first seeded onto permeable membrane inserts as a submerged model. Once confluent, the apical medium is removed, exposing the cells to air on the apical side while maintaining contact with differentiation culture medium in the basolateral domain (Bals et al., 2004; Braakhuis et al., 2020; Jiang et al., 2018). These environmental conditions drive differentiation into a pseudostratified epithelial phenotype (Jiang et al., 2018). The permeable inserts used for seeding, growth, and differentiation of ALI cultures are available with different pore densities (Corning Incorporated, 2007; Merck KGaA, 2024). Differences in ALI growth and differentiation conditions can affect the reproducibility of the HBEC model. This issue becomes especially relevant when supply chain disruptions require the use of alternative inserts. In this study, we cultured and differentiated primary HBEC on low pore density (LPD) and high pore density (HPD) membrane inserts to illustrate an approach for characterizing ALI culture differentiation through morphological and histological analyses.

## 2 MATERIALS AND METHODS

### 2.1 Primary bronchial epithelial cell culture

Primary HBEC were isolated from seven participants with no known history of asthma or chronic obstructive pulmonary disease undergoing bronchoscopies following written informed consent approved by the University of British Columbia and Providence Health Care Research Ethics Board (Approval number: H11‐02713 and H15‐02166). Bronchoscopy was performed using cytology brushes (Primed Canada, catalog #4206) to collect bronchial epithelial cells from small airways (<2 mm in diameter), generally in the right upper or left upper lobes. The mean age and standard deviation of the donors were 61.5 ± 20.5 years with a 3:4 male: female ratio, 2 nonsmokers, 3 former smokers, and 2 current smokers. Patient‐derived HBEC were dislodged into Pneumacult‐Ex medium (STEMCELL Technologies, catalog #05008) through gentle agitation and swirling of the brush into the media. Media was replaced every 2–3 days. After 14–16 days of culturing, expanded primary cells at >80% confluence were seeded as a submerged culture onto 0.4 μm pore size permeable membrane inserts in PneumaCult‐Ex Plus media (STEMCELL Technologies, catalog #05030). One ALI culture was generated per donor, and all were cultured and processed at the same time. Detachment was performed using Animal Component‐Free Cell Dissociation kit (STEMCELL Technologies, catalog #05426) and the cell density was 3 × 10^5^ cells/cm^2^. For the comparative evaluation, collagen type‐IV derived from human placenta (Millipore Sigma, catalog #C5533) was coated at 5 μg/cm^2^ on 24‐well polyethylene terephthalate (PET) membrane inserts with different pore densities: LPD insert with 4 × 10^6^ pores/cm^2^ (Corning Incorporated, catalog #3470) and a HPD insert with 1 × 10^8^ pores/cm^2^ (Millipore Sigma, catalog #PTHT24H48). For each primary HBEC sample, we established two ALI cultures: one on a LPD insert and one on a HPD insert. When the cells reached 90% confluence, the culture was air‐lifted, where the apical media was removed and basal media was replaced with complete PneumaCult‐ALI media (STEMCELL Technologies, catalog #05001) complete with kit supplements. The basal ALI medium was changed every 2 days, and the apical surface was rinsed weekly with warm Dulbecco's phosphate‐buffered saline (D‐PBS, Millipore Sigma, catalog #8662) to remove excess mucus.

### 2.2 Histology and morphological analysis

After 28 days of differentiation, ALI cultures were processed into formalin‐fixed paraffin‐embedded (FFPE) blocks. Fixation was performed in 10% normal buffered formalin (VWR, catalog #166004‐128) at 4°C overnight. The fixed tissues were processed using an automated tissue processor (Leica Biosystems, catalog #ASP6025) and subsequently embedded in paraffin (Fisher Scientific, catalog #AC171402500). Four micron‐thick sections were used for downstream histology and immunohistochemistry analysis. Hematoxylin and eosin (H&E) staining was performed for histological assessment and periodic acid‐Schiff (PAS) staining was done to detect polysaccharides abundant in mucus (Meyerholz et al., 2018). Five representative images taken near the centre of the ALI culture under light microscopy were taken at 400× magnification using the SPOT Advanced Imaging software (Diagnostic Instruments, RRID:SCR_016613). These images were analyzed using ImageJ, an image analysis software (National Institutes of Health, RRID:SCR_003070). For each image, mean epithelial thickness and mean cilia height, together with the total nuclear count per unit length of the basement membrane (TNC/BM), were calculated for each image. Five images were analyzed per ALI culture, and the values from these images were averaged to obtain representative measurements for each donor ALI. For mean ALI thickness, the area encompassed by the ALI was quantified and then divided by the length of the basement membrane of the ALI. To measure average cilia height, the apical domain area encompassed by cilia was quantified then divided by the apical length. To measure TNC/BM, individual nuclei were counted for each representative image and normalized to the length of the ALI basement membrane. The PAS signal was measured and normalized to the ALI area. Two independent, blinded observers analyzed the images.

### 2.3 Immunohistochemistry

Markers of airway epithelial differentiation and cell identity were probed using the Leica Bond RX automated immunohistochemistry stainer (Leica Biosystems, catalog #ASP6025). Antibodies used included mouse anti‐Cytokeratin 5 (KRT‐5) antibody (1 μg/mL, ab181491, Abcam, Cambridge, UK, RRID:AB_3678526), mouse anti‐Forkhead box protein J1 (FOXJ1) antibody (1 μg/mL, AMAb91254, Atlas Antibodies, Stockholm, Sweden, RRID:AB_2665866), rabbit anti‐Mucin 5B (MUC5B) antibody (0.2 μg/mL, HPA008246, Sigma‐Aldrich, RRID:AB_1854203), and mouse anti‐β‐tubulin IV antibody (1 μg/mL, MA5‐25559, Invitrogen, RRID:AB_2722895). Detection of these primary antibodies was performed using the BOND Polymer Refine Red Detection System (Leica Biosystems, catalog #DS9390). Nuclei counterstaining with hematoxylin is part of the BOND Polymer Detection System (mentioned above). Five representative images were taken at 400× magnification. Using ImageJ image analysis software, the ALI region of interest was manually traced, images were deconvoluted by color, made binary, and the target signal intensity was quantified and normalized to the ALI area and basement membrane length. Manual counting of KRT‐5 and FOXJ1 positive cells was performed by two blinded observers and averaged.

### 2.4 Transepithelial electrical resistance (TEER) measurements

Following 28 days of differentiation, the barrier function of HBEC‐derived ALI cultures was assessed by TEER measurements. TEER is the inverse of passive ion conductance from the apical to basal compartments of the permeable inserts. Differentiated airway epithelial cultures have higher TEER readings reflecting a functional epithelial barrier (Srinivasan et al., 2015). These measurements were recorded using the EVOM1 voltohmmeter (World Precision Instruments), repeated three times for each culture, and normalized to insert area (Ω•cm^2^).

### 2.5 Statistical analysis

Data were summarized and, for paired two group comparisons. Student's *t‐*tests were performed using Prism 9 (GraphPad Software, San Diego CA, USA, RRID:SCR_002798). Normal distribution of the data was assessed using the Shapiro–Wilk test, and a paired *t‐*test was used when the data were appropriately normal; otherwise, a Wilcoxon signed‐rank test was used. Statistical significance is set at *p* < 0.05.

## 3 RESULTS

**FIGURE 1:**
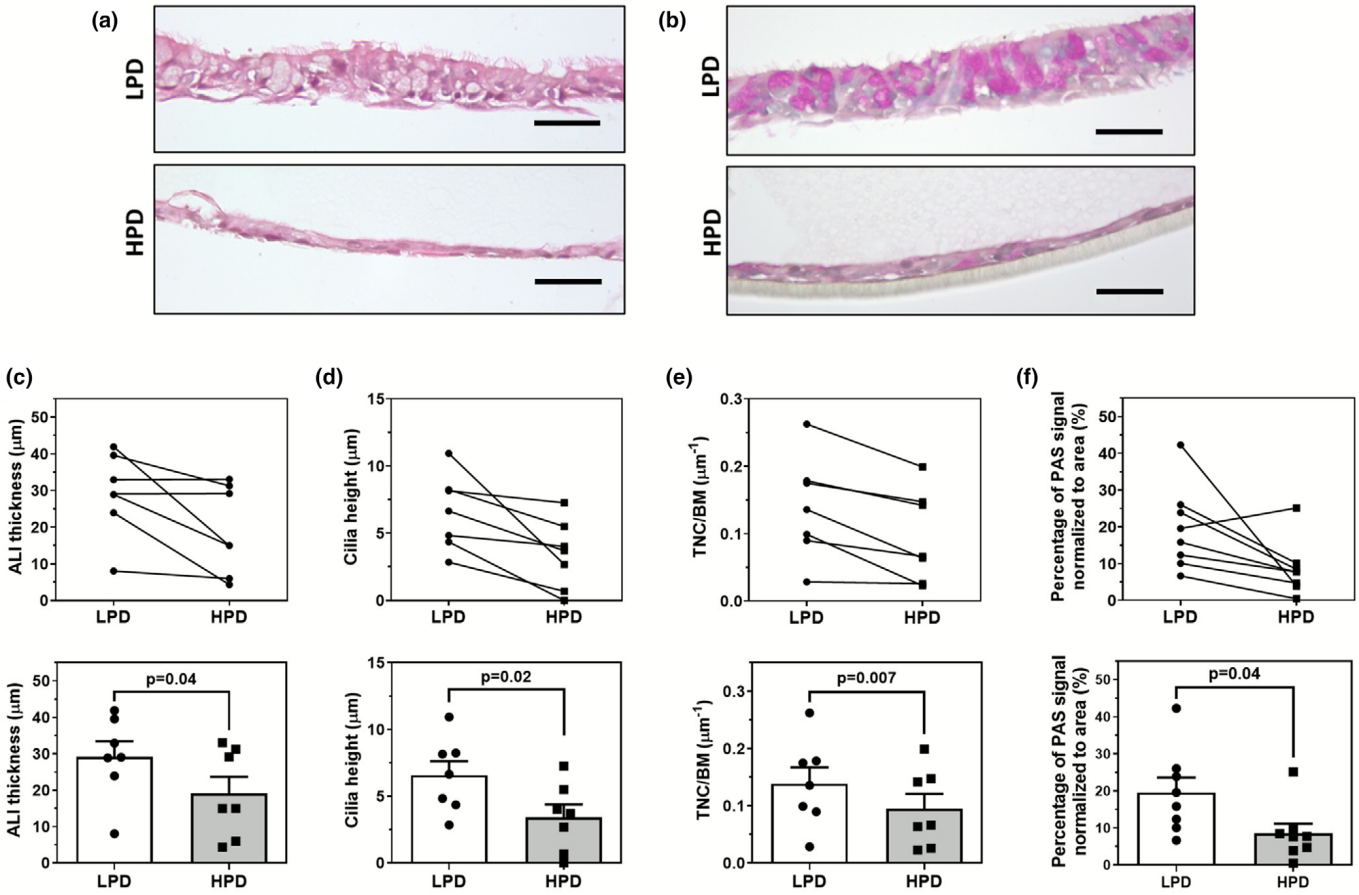
Growth of primary ALI cultures on permeable inserts of differing pore densities alters morphological measurements and polysaccharide expression. Representative images of hematoxylin and eosin (H&E) (a) and Periodic acid‐Schiff (PAS) stained (b) cross‐sections of 28 day old differentiated ALI culture grown on either a LPD or HPD permeable inserts. (c) Summary of mean ALI thickness per image, with 5 images obtained per ALI respectively for LPD and HPD inserts. (d) Summary of mean cilia height per image, with 5 images obtained per ALI, or the average distance from the base to the terminal end of a cilium. (e) Summary of total nuclei per μm of basement membrane (TNC/BM), which involves counting all nuclei in each image of ALI culture taken and normalized to the length of basement membrane. (f) PAS signal was quantified through normalizing area positive for PAS staining to the total ALI area. For all figures, the scale bar = 50 μm. Each datapoint represents the summarized measurement from 5 representative images from one independent donor ALI with one ALI generated per donor, *n* = 7. Data are presented as means ± SEM. Normality was assessed using the Shapiro–Wilk test; paired Student's *t*‐tests were applied to normally distributed data, and Wilcoxon signed‐rank tests were used otherwise.

HBEC‐derived ALI cultures were established on low and high pore density inserts, and we report findings from morphological and histological analyses used to characterize their differentiation. The translucency of HPD inserts made it difficult to monitor confluency and culture condition through light microscopy (data not shown). Therefore, ALI cultures grown on transparent LPD inserts were used as a visual proxy to determine when to air‐lift the culture. Following 28 days of differentiation, representative images of H&E stained cross sections show ALI grown on LPD or HPD inserts having different pseudostratified morphology (Figure 1a). ALI cultures grown on LPD inserts, compared to HPD, were significantly thicker (Figure 1c). LPD cultures also had significantly longer cilia (Figure 1d) as well as denser and more abundant cilia. Through manual cell counting, LPD ALI cultures demonstrated significantly more nuclei per unit of basement membrane than HPD ALI cultures (Figure 1e). PAS staining was localized to the vacuoles of non‐ciliated goblet cells within the ALI cultures (Figure 1b). PAS stained LPD ALI cultures also contained a significantly greater polysaccharide staining signal compared to HPD cultures (Figure 1f). The values of these morphological measurements are provided in Table 1.

**TABLE 1:** Morphological measurements of primary HBEC grown on low and high pore density permeable inserts.

	LPD	HPD	*p* Value
ALI height (μm)	29.2 ± 4.3	19.1 ± 4.5	0.04
Cilia height (μm)	6.6 ± 1.0	3.4 ± 1.0	0.02
Total nuclear count/basement membrane length (μm^−1^)	0.139 ± 0.029	0.095 ± 0.067	0.007
PAS staining signal (%)	19.6 ± 4.0	8.5 ± 2.6	0.04

*Note*: Data are presented as mean ± SEM with 7 independent donors for each insert condition. Shapiro–Wilk test was used to test for normality; paired Student's *t*‐tests were applied to normally distributed data, and Wilcoxon signed‐rank tests were used otherwise.

**FIGURE 2:**
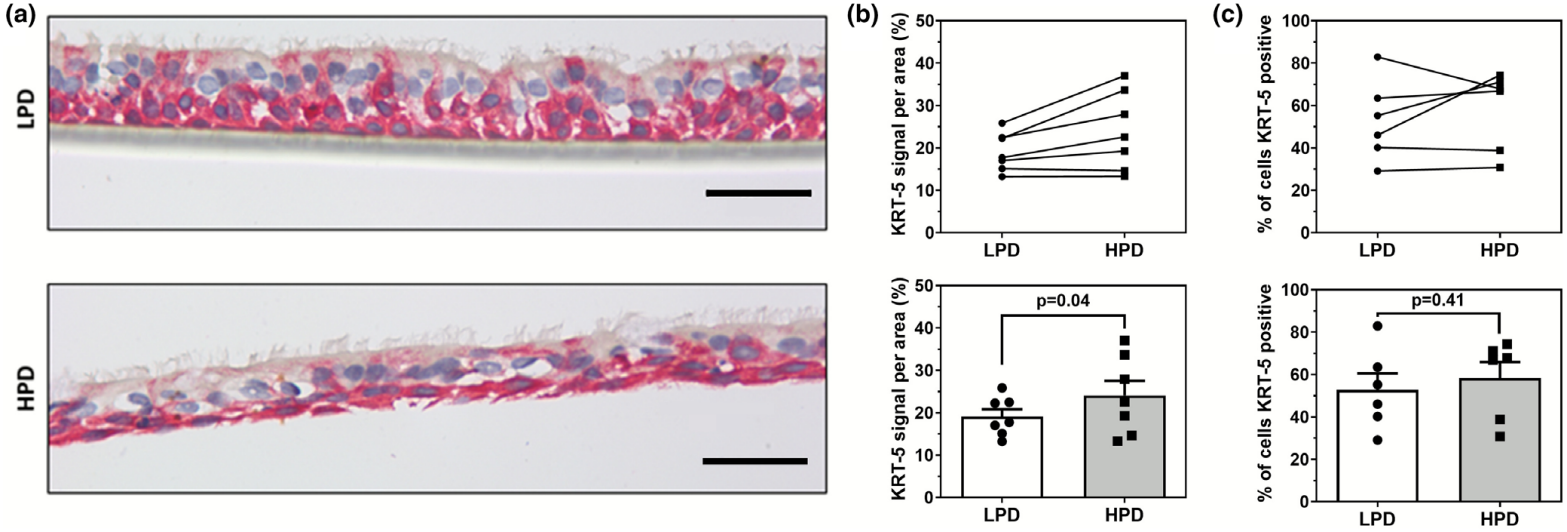
KRT‐5 expression is altered in primary HBEC ALI cultures grown on LPD and HPD inserts. (a) Representative images of ALI cultures grown on LPD and HPD inserts, immunostained with a mouse anti‐cytokeratin 5 antibody and counterstained with hematoxylin. (b) Color segmentation image analysis of KRT‐5 immunostained ALI sections shows significantly increased expression normalized to the ALI area. (c) Manual cell counting for KRT‐5 positive cells demonstrates no difference in proportion between ALI cultures grown on LPD and HPD inserts. For all figures, the scale bar = 50 μm. Each datapoint represents the summarized measurement from 5 representative images from one independent donor ALI with one ALI generated per donor, *n* = 7. Data are presented as means ± SEM. Normality was assessed using the Shapiro–Wilk test; paired Student's *t*‐tests were applied to normally distributed data, and Wilcoxon signed‐rank tests were used otherwise.

The expression of cell‐type‐specific markers, starting with KRT‐5, a marker of basal cells which serve as progenitor stem cells crucial for airway homeostasis and repair (Wu et al., 2022), was characterized. We observed increased KRT‐5 staining in the apical domain of ALI cultures grown on LPD inserts. In contrast, KRT‐5‐expressing cells were more localized to the basal domain in cultures grown on HPD inserts (Figure 2a). The expression of KRT‐5 was significantly greater in ALI cultures grown on HPD than LPD inserts (Figure 2b). In donors with comparatively higher KRT‐5 expression on LPD inserts, this expression was further enhanced in ALI cultures on HPD inserts. However, there was no significant difference in the percentage of cells positive for KRT‐5 between HPD and LPD inserts (Figure 2c).

**FIGURE 3:**
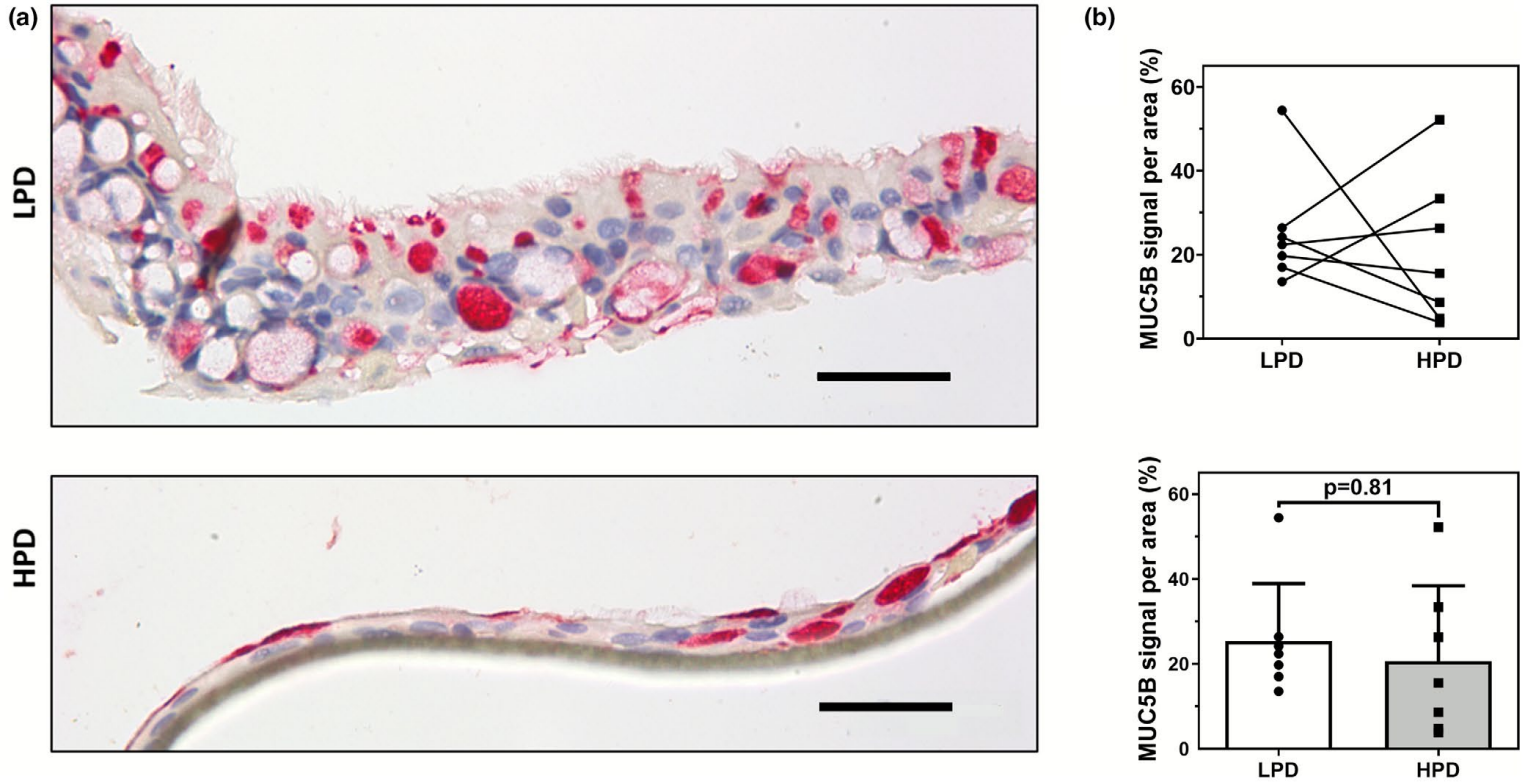
MUC5B expression is altered in primary HBEC ALI cultures grown on LPD and HPD inserts. (a) Representative images of ALI cultures grown on LPD and HPD inserts, immunostained with a rabbit anti‐MUC5B antibody, and counterstained with hematoxylin. (b) Color segmentation image analysis of MUC5B immunostained ALI sections normalized to the ALI area was summarized. For all figures, the scale bar = 50 μm. Each datapoint represents the summarized measurement from 5 representative images from one independent donor ALI with one ALI generated per donor, *n* = 7. Data are presented as means ± SEM. Normality was assessed using the Shapiro–Wilk test with a Wilcoxon signed‐rank test used here.

The expression of MUC5B, one of the main secreted airway mucins, was localized to the vacuoles and cytoplasm of goblet cells within the ALI cultures (Figure 3a). There was no significant difference in the expression of MUC5B between ALI cultures grown on LPD or HPD inserts (Figure 3b).

**FIGURE 4:**
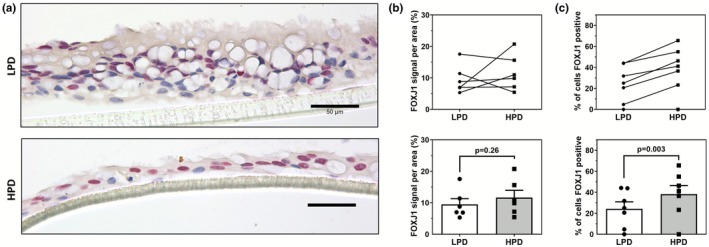
FOXJ1 expression is altered in primary HBEC ALI cultures grown on LPD and HPD inserts. (a) Representative images of ALI cultures grown on LPD and HPD inserts, immunostained with a mouse anti‐FOXJ1 antibody, and counterstained with hematoxylin. (b) Color segmentation image analysis of FOXJ1 immunostained ALI sections shows no significant difference in expression between ALI cultured on LPD versus HPD inserts. (c) Manual cell counting for FOXJ1 positive cells demonstrates a significant difference in proportion between ALI cultures grown on LPD and HPD inserts. For all figures, the scale bar = 50 μm. Each datapoint represents the summarized measurement from 5 representative images from one independent donor ALI with one ALI generated per donor, *n* = 7. Data are presented as means ± SEM. Normality was assessed using the Shapiro–Wilk test with a paired Student's *t*‐test used here.

The expression of FOXJ1, a transcription factor that controls ciliogenesis and a ciliated cell‐specific marker, was found localized to the nuclei of ciliated cells within the ALI cultures (Figure 4a).

Although there was no significant difference in FOXJ1 expression between ALI cultures grown on LPD or HPD inserts (Figure 4b), ALI cultures grown on HPD inserts showed a significantly greater proportion of FOXJ1 positive compared to cultures grown on LPD inserts (Figure 4c).

**FIGURE 5:**
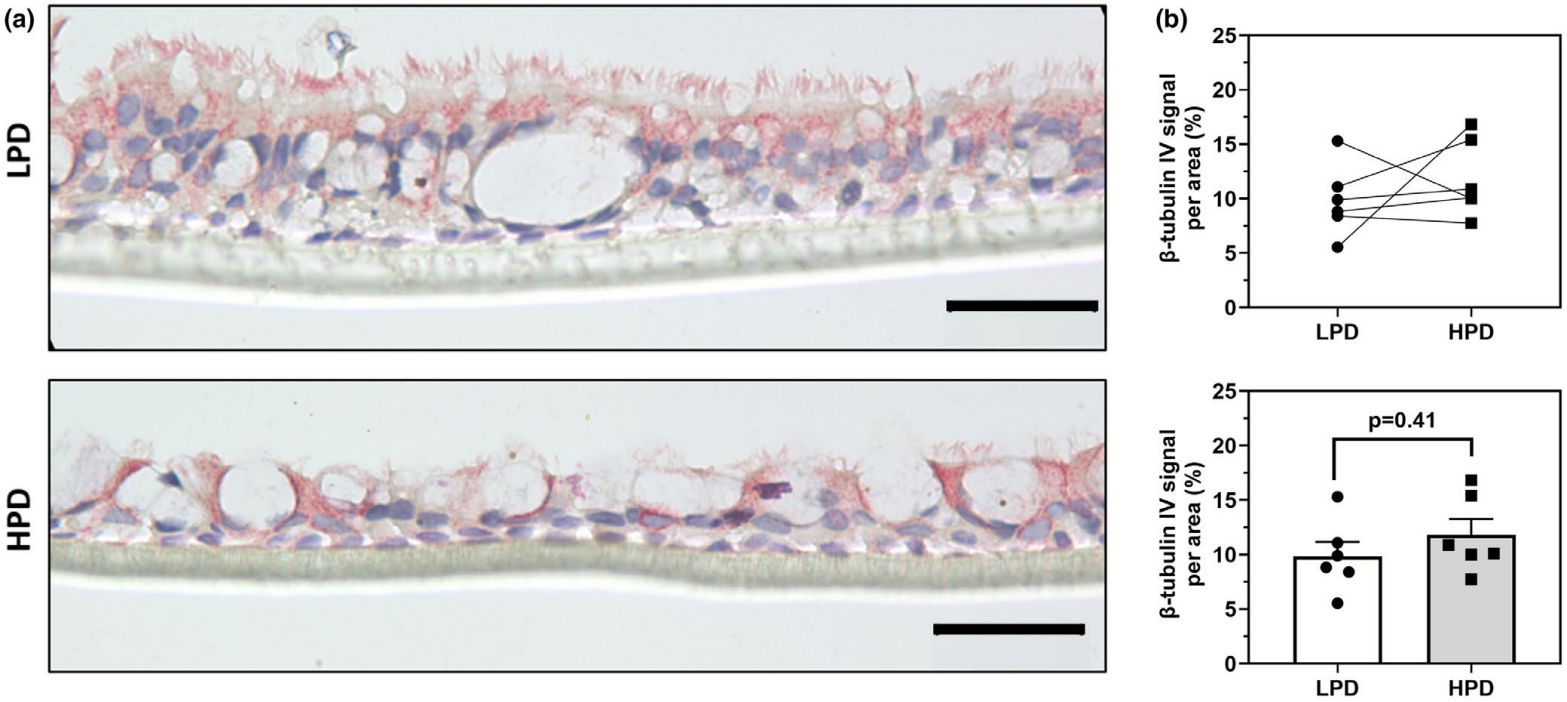
β‐tubulin‐IV expression is not significantly altered in primary HBEC ALI cultures grown on LPD and HPD inserts. (a) Representative images of ALI cultures grown on LPD and HPD inserts and immunostained with a mouse anti‐β‐tubulin‐IV antibody and counterstained with hematoxylin. (b) Color segmentation image analysis of β‐tubulin‐IV immunostained ALI sections shows no significant difference in expression between ALI grown on LPD versus HPD inserts. (c) Manual cell counting for β‐tubulin‐IV positive cells demonstrates no difference proportion between ALI cultures grown on LPD and HPD inserts. For all figures, the scale bar = 50 μm. Each datapoint represents the summarized measurement from 5 representative images from one independent donor ALI with one ALI generated per donor, *n* = 7. Data are presented as means ± SEM. Normality was assessed using the Shapiro–Wilk test with a paired Student's *t*‐test used here.

Expression of β‐tubulin IV, one of the main tubulin isotypes within airway cilia, was localized to the cilia and diffusely through the cytoplasm (Figure 5a). Its expression was not significantly different between ALI grown on LPD versus HPD inserts (Figure 5b). The values of our characterization of cell‐type specific markers are provided in Table 2.

**TABLE 2:** Summary of immunohistochemical staining of primary HBEC ALI cultures grown on LPD and HPD permeable inserts.

	LPD	HPD	*p* Value
Cytokeratin 5 (KRT‐5)			
Staining signal (%)	19.1 ± 1.7	24.1 ± 3.5	0.04
% cells positive	52.9 ± 7.7	58.3 ± 7.6	0.41
Mucin 5B (MUC5B)			
Staining signal (%)	25.4 ± 5.1	20.7 ± 6.7	0.64
Forkhead box J1 (FOXJ1)			
Staining signal (%)	8.3 ± 1.9	11.6 ± 2.0	0.26
% cells positive	24.3 ± 6.6	38.3 ± 8.2	0.003
β‐tubulin‐IV			
Staining signal (%)	9.8 ± 1.3	11.8 ± 1.4	0.42

**FIGURE 6:**
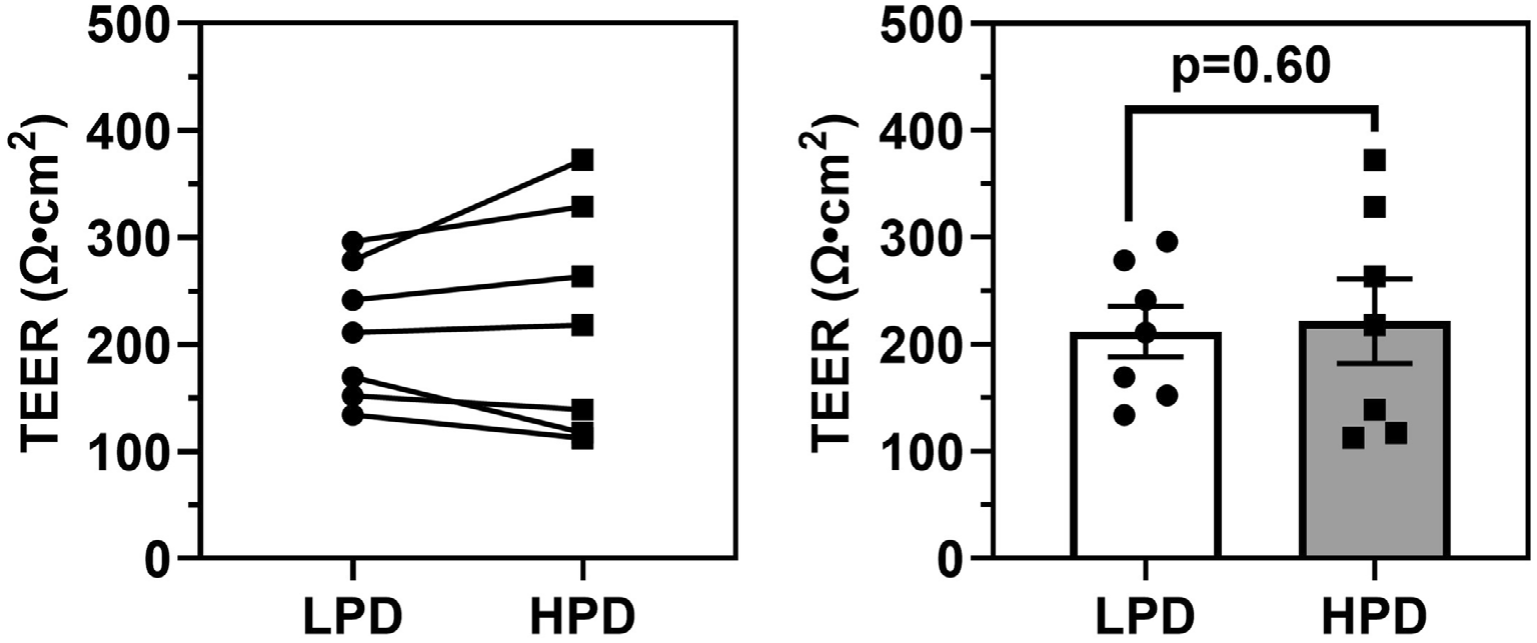
Transepithelial electrical resistance is not significantly different between ALI cultures grown on LPD and HPD inserts. Following 28 days of differentiation, three TEER measurements for each of primary ALI cultures grown on LPD and HPD inserts were taken. The three observations were averaged and are displayed by a dot, with each datapoint representing an independent donor, *n* = 7. Normality was assessed using the Shapiro–Wilk test, with a paired Student's *t*‐test used here with no significant difference in TEER between LPD and HPD.

TEER, a functional measurement of epithelial barrier integrity, was obtained from all cultures grown on LPD and HPD inserts following 28 days of differentiation (Figure 6). TEER was not significantly different between LPD and HPD inserts (211.8 ± 23.8 vs. 221.7 ± 39.5 Ω·cm^2^, *p* = 0.60).

## 4 DISCUSSION

The use of porous permeable membranes is crucial for differentiating airway epithelial tissues with distinct apical and basolateral domains. After the epithelial cultures reach confluence, removal of the apical medium increases oxygenation to promote differentiation (Bebök et al., 2001). The culture maintains contact with the basal medium through the membrane, which aims to replicate the structure of the airway epithelium and extracellular space in vitro (Cao et al., 2021). Under these biphasic conditions, the permeable membrane insert, which serves as the interface between the basal medium and the culture, is therefore essential for the growth and differentiation of the culture. These inserts facilitate the diffusion of small molecules, including nutrients and growth factors, across the membrane, which can be influenced by factors such as pore size and density (Chung et al., 2018).

In this study, we present a method to characterize the differentiation of ALI cultures using morphological, histological, and functional analyses. Our findings show that the growth of ALI cultures on inserts of differing pore density can change the morphological and differentiation characteristics of these cultures. We observed that ALI cultures grown on LPD inserts were thicker, had longer cilia, and had more cells per unit length of the culture. Furthermore, PAS staining revealed greater mucus secretion on ALI cultures grown on LPD inserts than HPD inserts. HPD inserts were observed to have a similar pseudostratified epithelial morphology but with shorter and fewer cilia and less polysaccharide content. These observations suggest that pore density can alter the morphology of the ALI cultures. ALI cultures grown on HPD inserts, which have shorter and less dense cilia, can resemble epithelia affected by cigarette smoke exposure and COPD. These conditions are associated with alterations in cilia structure and function, leading to impaired mucociliary clearance due to changes in intraflagellar transport gene expression, epithelial differentiation, and oxidative stress (Hessel et al., 2014; Lam et al., 2013; Schamberger et al., 2015). However, mucin expression and mucus content within the airways, which correlates with PAS staining (Meyerholz et al., 2018), is increased in COPD small airways, associated with the accumulation of inflammatory mucus exudates and goblet cell hyperplasia (Innes et al., 2006; Kim et al., 2015; Hogg et al., 2004).

Our findings contrast with those of Cozens et al., who reported that bovine bronchial epithelial cells differentiated on LPD inserts developed into non‐ciliated squamous epithelia, whereas cells grown on HPD inserts were well‐differentiated. They observed that epithelia cultured on HPD inserts exhibited greater thickness, a higher degree of ciliation, and increased mucus secretion compared to those on LPD inserts. However, several factors could explain these differences. First, their use of bovine epithelial cells may introduce species‐specific differences in epithelial differentiation potential, as comparative studies utilizing bovine and human airway cultures are sparse. Second, they utilized an in‐house submerged growth medium composed of DMEM and Ham's F‐12, which differs from the experimental protocol outlined in our study which used the PneumaCult‐ALI medium (Cozens et al., 2018). Prior studies have demonstrated that media composition significantly affects ALI morphology, differentiation, and gene expression profiles (Awatade et al., 2023; Leung et al., 2020; Saint‐Criq et al., 2020), potentially altering cellular responses to different pore densities. Thus, further investigation is needed to determine if variations in cell origin and culture medium contribute to the observed differences.

We observed greater KRT‐5 signal but no change in the proportion of KRT‐5 positive cells in ALI cultures on HPD compared to LPD inserts. Additionally, the apical KRT‐5 signal was more prominent in LPD inserts. As basal cells move upward from the basal lamina, lose quiescence, proliferate, and differentiate towards a columnar phenotype, KRT‐5 expression is gradually reduced (Alam et al., 2011; Bukowy‐Bieryłło et al., 2022; Ruysseveldt et al., 2021). This suggests that in ALI cultures on LPD inserts, basal cells may be more active in constituting the epithelial culture, whereas in HPD inserts, the basal population is more quiescent. The proportion of FOXJ1 positive cells is greater in ALI cultures on HPD inserts, despite a higher total nuclear count per basement membrane in LPD cultures. This suggests that HPD inserts may promote a higher proportion of ciliated cell differentiation relative to other epithelial cell types or due to the higher TNC/BM observed in LPD cultures which is also associated with a higher proportion of non‐FOXJ1 positive cells. Furthermore, TEER measurements between ALI on LPD or HPD inserts were not significantly different, indicating similar barrier integrity between the two.

Brocke et al. compared TEER, expression of genes associated with mucin production, motile cilia, and inflammatory markers, ciliary beat frequency, and ciliated area of human nasal epithelial cells grown and differentiated on CELLTREAT® and Corning® inserts. The pore size and material composition of the inserts from both manufacturers were similar, with pore densities of 2 × 10^6^ pores/cm^2^ for CELLTREAT® and 4 × 10^6^ pores/cm^2^ for Corning® inserts. They found no significant differences between the cultures grown on either type of insert, concluding that these inserts are comparable and likely do not impact cellular differentiation (Brocke et al., 2024). However, unlike Brocke et al.'s study, which used inserts with relatively similar pore densities and found no significant impact on differentiation, our study reveals that larger differences in pore density can elicit notable changes in the differentiation attributes of ALI cultures.

Limitations of this study include that only two specific pore densities were examined as intermediate densities such as 2 × 10^6^ pores/cm^2^ being available (Sarstedt AG & Co, n.d.; Corning Incorporated, 2007). Future studies should explore a broader range of pore densities to fully understand how their impact on epithelial differentiation. Additionally, donor variability, encompassing differences in age, smoking history, and other demographic and clinical factors, may have influenced the results, potentially obscuring specific effects of pore density. Notably, there is a negative association between TEER and age, with lower TEER observed in subjects older than 45 years of age compared to those younger (de Vries et al., 2022). Although morphological studies of ALI cultures derived from smokers and nonsmokers are limited, in vitro cigarette smoke exposure has been observed to alter ALI differentiation (Beisswenger et al., 2004; Gindele et al., 2020; Schamberger et al., 2015). Lastly, though TEER was performed to measure tightness of barrier to ion flow, baseline assessment of junctional proteins, especially zonula occludens‐1 found in tight junctions (Bartosova et al., 2021), was not performed. TEER can be influenced by cell morphology, junctional length, and presence of immature junctions which may not directly correlate with the expression and localization of tight junction proteins (Felix et al., 2021). Together, these considerations suggest that variation in ALI differentiation outcomes can arise from practical, study‐specific choices in culture setup, such as insert pore density, and may therefore be expected across laboratories. In this context, studies using ALI cultures may benefit from applying a consistent set of measurements to assess and confirm epithelial differentiation, incorporating epithelial morphology, cilia structure, barrier integrity, cell‐type–specific marker expression, and functional responsiveness to relevant stimuli. Several of these features are captured by the approach described here, which integrates morphological, histological, molecular, and functional analyses to provide a scalable framework for quantitatively linking epithelial structure, function, and cell‐type composition, with the goal of improving reproducibility across studies.

## 5 CONCLUSION

Permeable membrane insert pore density can impact the morphological measurements and airway epithelial markers of air‐liquid interface cultures derived from primary human bronchial epithelial cells. Careful selection of permeable inserts is necessary depending on the experimental goals, intended analyses, and the specific characteristics of the epithelial cultures desired. This consideration is especially relevant during supply chain disruptions. Our data highlight that substitutions can introduce variability in outcomes, underscoring the need to standardize these in vitro cultures and additional validation to ensure data reproducibility across studies.

## AUTHOR CONTRIBUTIONS

Conceptualization, T.G., G.K.S., and D.D.; experimentation and data acquisition, T.G., G.K.S.; data analysis, T.G., J.M.; writing—original draft preparation, T.G.; writing, review, and editing, T.G., G.K.S., J.M., W.Y.L., J.L., and D.D.; supervision, G.K.S., D.D.; funding acquisition, J.L. and D.D. All authors have read and agreed to the published version of the manuscript.

## FUNDING INFORMATION

This work was funded by the Providence Healthcare Research Institute and St. Paul's Foundation. T.J.F.G. is supported by an award jointly funded by Asthma Canada, the Canadian Allergy, Asthma, and Immunology Foundation (CAAIF). He is also supported by the Canadian Graduate Scholarships—Master's Program funded by the CIHR and the University of British Columbia MD/PhD Studentship. G.K.S., J.M.V., and W.Y.L. have no funding to disclose. J.M.L. is a Tier 2 Canada Research Chair in Translational Airway Biology. D.R.D. acknowledges the CIHR, Michael Smith Health Research BC, and the BC Lung Foundation for their support.

## CONFLICT OF INTEREST STATEMENT

The authors declare no conflict of interest. The funders had no role in the design of the study, in the collection, analyses, or interpretation of data, in the writing of the manuscript, or in the decision to publish the results.

## ETHICS STATEMENT

Before starting this study, the University of British Columbia and the Providence Health Care Research Ethics Board reviewed and approved the study. Approval number: H11‐02713 and H15‐02166.

## INFORMED CONSENT STATEMENT

Informed consent was obtained from all subjects involved in the study prior to their bronchoscopy procedure.

## Data Availability

All the collected relevant research data is presented in this paper. However, further inquiries can be directed to the corresponding authors.
